# Antibody and transcription landscape in peripheral blood mononuclear cells of elderly adults over 70 years of age with third dose of COVID-19 BBIBP-CorV and ZF2001 booster vaccine

**DOI:** 10.1186/s12979-023-00408-x

**Published:** 2024-01-27

**Authors:** Yuwei Zhang, Lianxiang Zhao, Jinzhong Zhang, Xiaomei Zhang, Shanshan Han, Qingshuai Sun, Mingxiao Yao, Bo Pang, Qing Duan, Xiaolin Jiang

**Affiliations:** 1 Infectious Disease Prevention and Control Section, Shandong Center for Disease Control and Prevention, Jinan, Shandong Province China; 2 School of Public Health and Management, Binzhou Medical University, Yantai , Shandong Province China; 3 Liaocheng Center for Disease Control and Prevention, Liaocheng, Shandong Province China; 4 School of Public Health and Health Management, Shandong First Medical University & Shandong Academy of Medical Sciences, Jinan, Shandong Province China; 5 Shandong Provincial Key Laboratory of Infectious Disease Control and Prevention, Shandong Center for Disease Control and Prevention, 16992 Jingshi Road , Jinan, 250014 Shandong Province China

**Keywords:** COVID-19, Third booster vaccine, Aging, Systems biology analysis, Antibody responses, RNA-seq, Transcriptome analysis

## Abstract

**Background:**

In the context of the COVID-19 pandemic and extensive vaccination, it is important to explore the immune response of elderly adults to homologous and heterologous booster vaccines of COVID-19. At this point, we detected serum IgG antibodies and PBMC sample transcriptome profiles in 46 participants under 70 years old and 25 participants over 70 years old who received the third dose of the BBIBP-CorV and ZF2001 vaccines.

**Results:**

On day 7, the antibody levels of people over 70 years old after the third dose of booster vaccine were lower than those of young people, and the transcriptional responses of innate and adaptive immunity were also weak. The age of the participants showed a significant negative correlation with functions related to T-cell differentiation and costimulation. Nevertheless, 28 days after the third dose, the IgG antibodies of elderly adults reached equivalence to those of younger adults, and immune-related transcriptional regulation was significantly improved. The age showed a significant positive correlation with functions related to "chemokine receptor binding", "chemokine activity", and "chemokine-mediated signaling pathway".

**Conclusions:**

Our results document that the response of elderly adults to the third dose of the vaccine was delayed, but still able to achieve comparable immune effects compared to younger adults, in regard to antibody responses as well as at the transcript level.

**Supplementary Information:**

The online version contains supplementary material available at 10.1186/s12979-023-00408-x.

## Background

In the third year of the fight against COVID-19, the World Health Organization declared that the COVID-19 pandemic no longer constitutes a Public Health Emergency of International Concern (PHEIC), but this does not mean that COVID-19 is over as a global health threat. To date, clinical experience has shown that COVID-19 is heterogeneous, ranging from asymptomatic and mild infection to severe and fatal disease. Aging itself is a prominent risk factor for severe disease and death from COVID-19 [1]. Given the high proportion of severe to critical cases and high fatality rate observed in elderly COVID-19 patients [2], COVID-19 has emerged as an emergent disease of aging [3].

Vaccines always work by tricking our immune system into developing "immune memory" mediated by B and T cells against specific infectious pathogens and are one of the most effective ways to prevent infection or reduce symptom severity [4]. With aging, the immune response undergoes dynamic remodeling, and immunosenescence occurs, which is a phenomenon of gradual deterioration of innate and adaptive immune responses [5]. Immunosenescence leads not only to increased susceptibility to infection but also to an impaired immune response to vaccination [6, 7]. With changing demographics around the globe, often described as "the gray tsunami", understanding the immune response of the elderly to homologous and heterologous COVID-19 booster vaccines is not an option, it is a necessity [8].

The application of high throughput sequencing technology combined with bioinformatics methods in systems biology analysis is ideal for studying the immune response mediated by viral infection and vaccination [9–12]. Previously, systems biology analyses of the BNT162b mRNA vaccine [13], influenza vaccine [14], and VSV-EBOV vaccine [15] have fully revealed the dynamics of the complex global immune response of the host after vaccination. Here, we employed IgG antibody detection and transcriptional profiling analyses in young (age < 70) and elderly (age ≥ 70) adults using blood samples following inactivated BBIBP-CorV and protein subunit ZF2001 booster vaccination. Our results revealed that 7 days after vaccination, elderly people exhibit dysregulation and damage related to functional pathways such as T-cell activation and differentiation, resulting in a delayed immune response to the COVID-19 vaccine booster. However, 28 days after vaccination, the cytokine-related functional pathways in the elderly were significantly activated, and antibody levels were similar to those in young adults.

## Results

### Characteristics of participants and samples

**Fig. 1:**
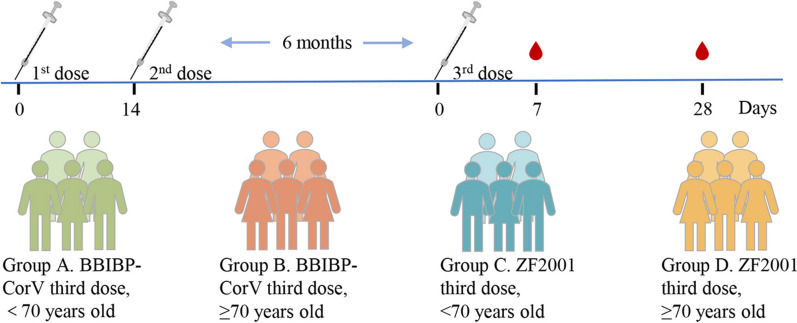
The details of this study design, including age characteristics of participants, booster vaccine type, and samples collection time in the 4 booster groups

A total of 71 participants who had completed two doses of primary immunization with the inactivated vaccine for more than 6 months were recruited to receive a third dose of either the BBIBP-CorV or ZF2001 vaccine. All participants were divided into four groups according to age and booster dose, including group A (boosted by BBIBP-CorV, < 70 years old), group B (boosted by BBIBP-CorV, ≥ 70 years old), group C (boosted by ZF2001, < 70 years old), and group D (boosted by ZF2001, ≥ 70 years old) (Fig. 1). At days 7 and 28 after vaccination, we collected a total of 131 serum and 57 PBMC samples. The detailed characteristics of the participants are shown in Table S1 and Table S2.

### Antibody response

**Fig. 2:**
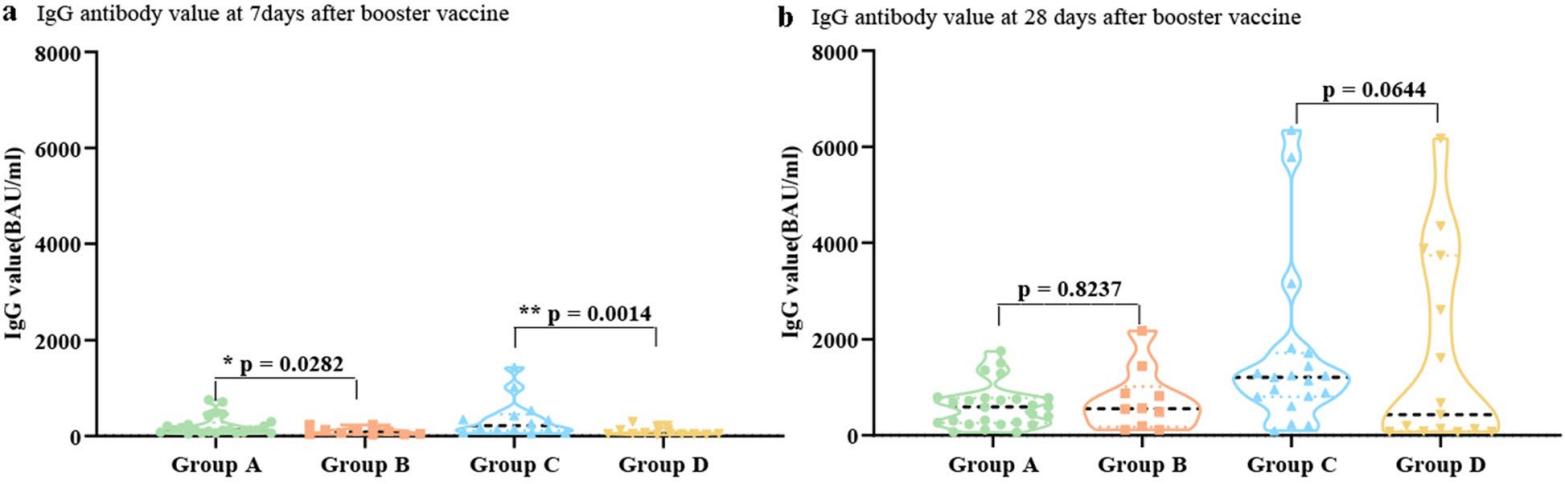
The level of IgG antibodies against SARS-CoV-2 of serum samples 7 (**a**) and 28 (**b**) days after booster vaccination. P values were computed by unpaired t-tests with log-transformation using GraphPad Prism 8.0

First, we estimated the titers of binding antibodies from sera collected at 7 and 28 days after vaccination of the participants. After 7 days of homologous boost with BBIBP-CorV, the mean value of IgG antibody in the younger group was 221.09 BAU/ml, and that in the elderly group was 104.35 BAU/ml. The elderly group was significantly lower than the younger group (*P* = 0.0282). A similar phenomenon was observed in different age groups heterologously boosted by ZF2001, with 366.63 BAU/ml in the younger group and 89.24 BAU/ml in the elder group, and the difference was statistically significant (*P* = 0.0014) (Fig. 2a). After 28 days of the third booster dose, the mean IgG antibody levels in the four groups increased to 610.6, 733.9, 1631, and 1617 BAU/ml, respectively. Remarkably, there was no age-associated difference in antibody responses(*P* = 0.8237, *P* = 0.0644) (Fig. 2b).

### Differential expression analysis of transcriptome profiles of participants with the third dose of COVID-19 booster vaccine

**Fig. 3:**
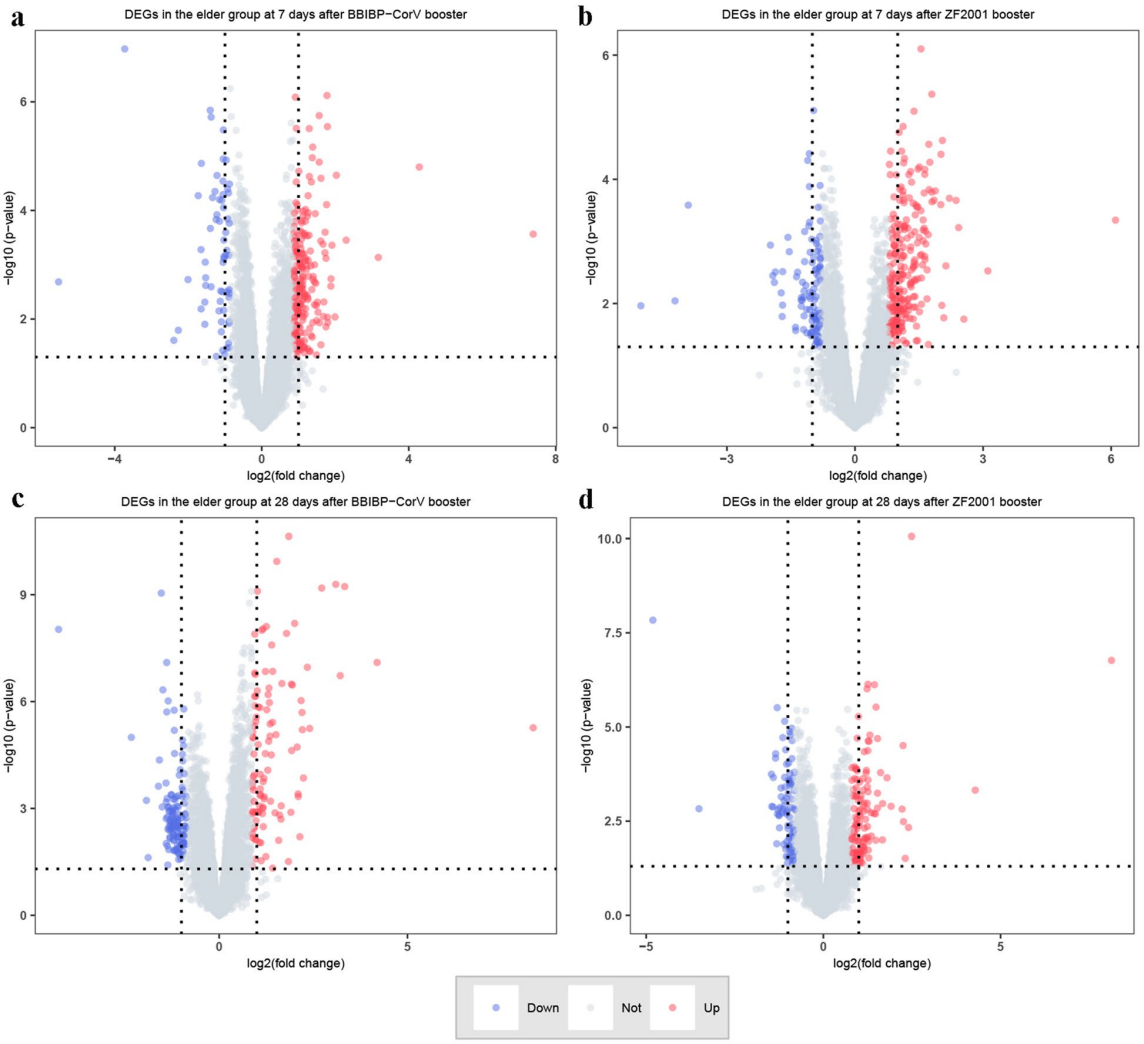
Volcano plot of global gene expression changes in the elderly group induced by the third dose of BBIBP-CorV (**a**) and ZF2001 (**b**) at 7 days. Volcano plot of global gene expression changes in the elderly group induced by the third dose of BBIBP-CorV (**c**) and ZF2001 (**d**) at 28 days. Red: up-regulated DEGs in the elderly group compared with the younger group; Blue: down-regulated DEGs in the elderly group compared with the younger group; Grey: non-DEGs. Criteria: |Fold Change|= 2^^logFC_cutoff^ is marked out by two black dotted lines. *P*-value = 0.05 is marked out by a horizontal black line y =  − Log_10_.^(0.05)^

**Fig. 4:**
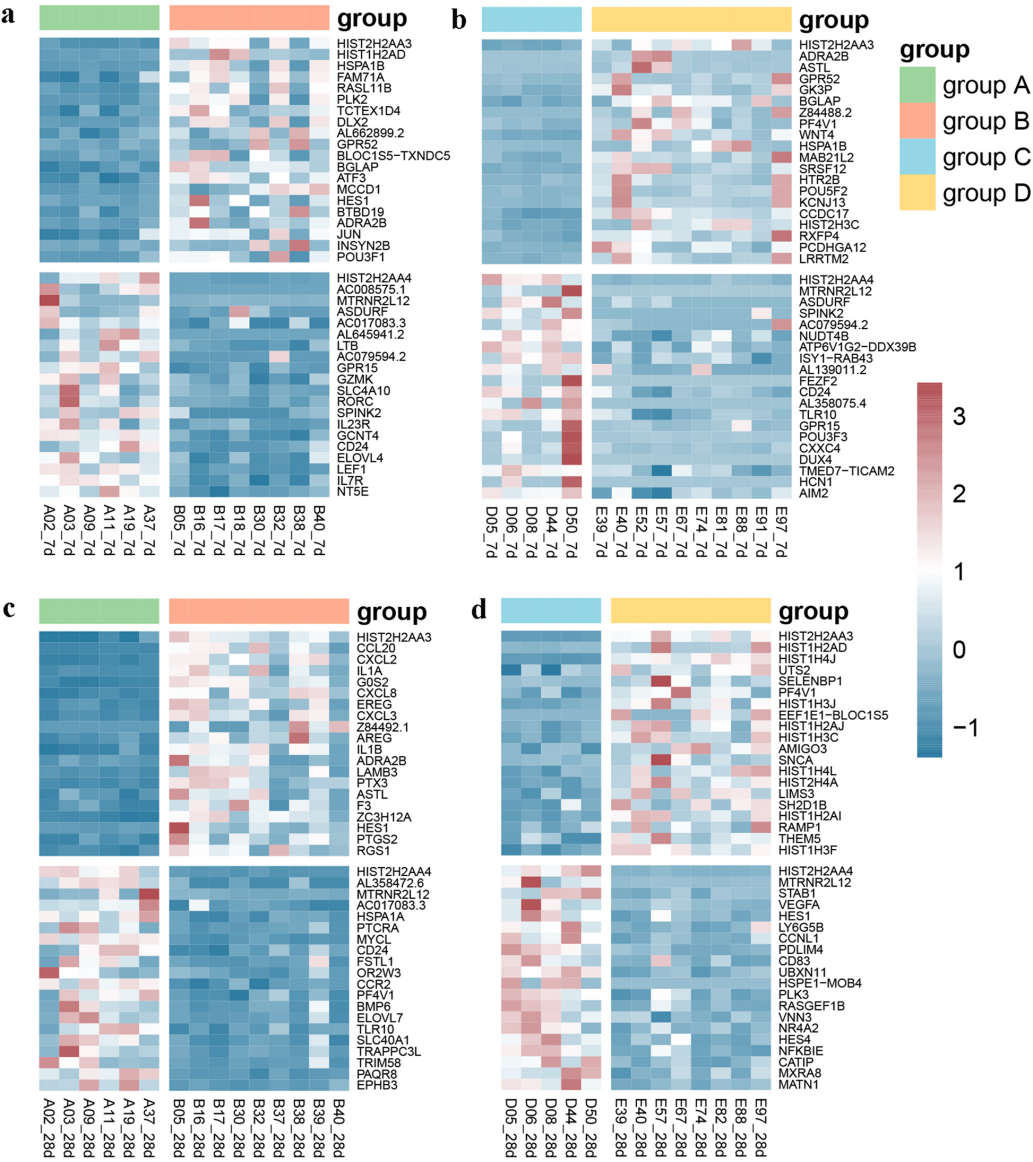
**a** The expression profile of the top 20 up-regulated and down-regulated DEGs in the elderly group compared with the younger group boosted by BBIBP-CorV after 7 days. **b** The expression profile of the top 20 up-regulated and down-regulated DEGs in the elderly group compared with the younger group boosted by ZF2001 after 7 days. **c** The expression profile of the top 20 up-regulated and down-regulated DEGs in the elderly group compared with the younger group boosted by BBIBP-CorV after 28 days. **d** The expression profile of the top 20 up-regulated and down-regulated DEGs in the elderly group compared with the younger group boosted by ZF2001 after 28 days. Each row represents mRNA and each column represents a sample. Red indicates higher expression and blue indicates low expression

To determine the effect of aging on the transcriptional program of the third dose of COVID-19 vaccine, we performed a transcriptome systematic scan of 57 PBMC samples from different groups. After 7 days of homologous boost with BBIBP-CorV, the number of up-regulated and down-regulated DEGs in the elder group compared with the younger group was 213 and 65, respectively(Fig. 3a). After 7 days of ZF2001 heterologous boost, 136 and 83 genes were up-regulated and down-regulated in the elder group compared with the younger group, respectively(Fig. 3b). The up-regulated DEGs of the elder group after 28 days with the BBIBP-CorV booster were 100, and the down-regulated DEGs were 134(Fig. 3c). There were 131 up-regulated DEGs and 84 down-regulated DEGs in the elder group after 28 days of the ZF2001 booster(Fig. 3d). We used the heatmap to show the expression of the top 20 up-regulated and down-regulated differentially expressed genes in each sample(Fig. 4a-d).

### Impairment of the immune transcriptional response to the vaccine in aging on Day 7

In order to analyze the interaction relationships between DEGs, the STRING database was used to create a PPI network. Using the MCODE plug-in of Cytoscape software, we extracted 4 subnetworks from the PPI network at the nodes and edges of all DEGs, including the characteristic induced up-regulated and depleted down-regulated DEG sets identified from the third dose of BBIBP-CorV(Figure S1a, b) and ZF2001(Figure S1c, d) in elderly individuals. These characteristic gene subnetworks contained 21, 25, 34, and 42 DEGs, respectively.

**Fig. 5:**
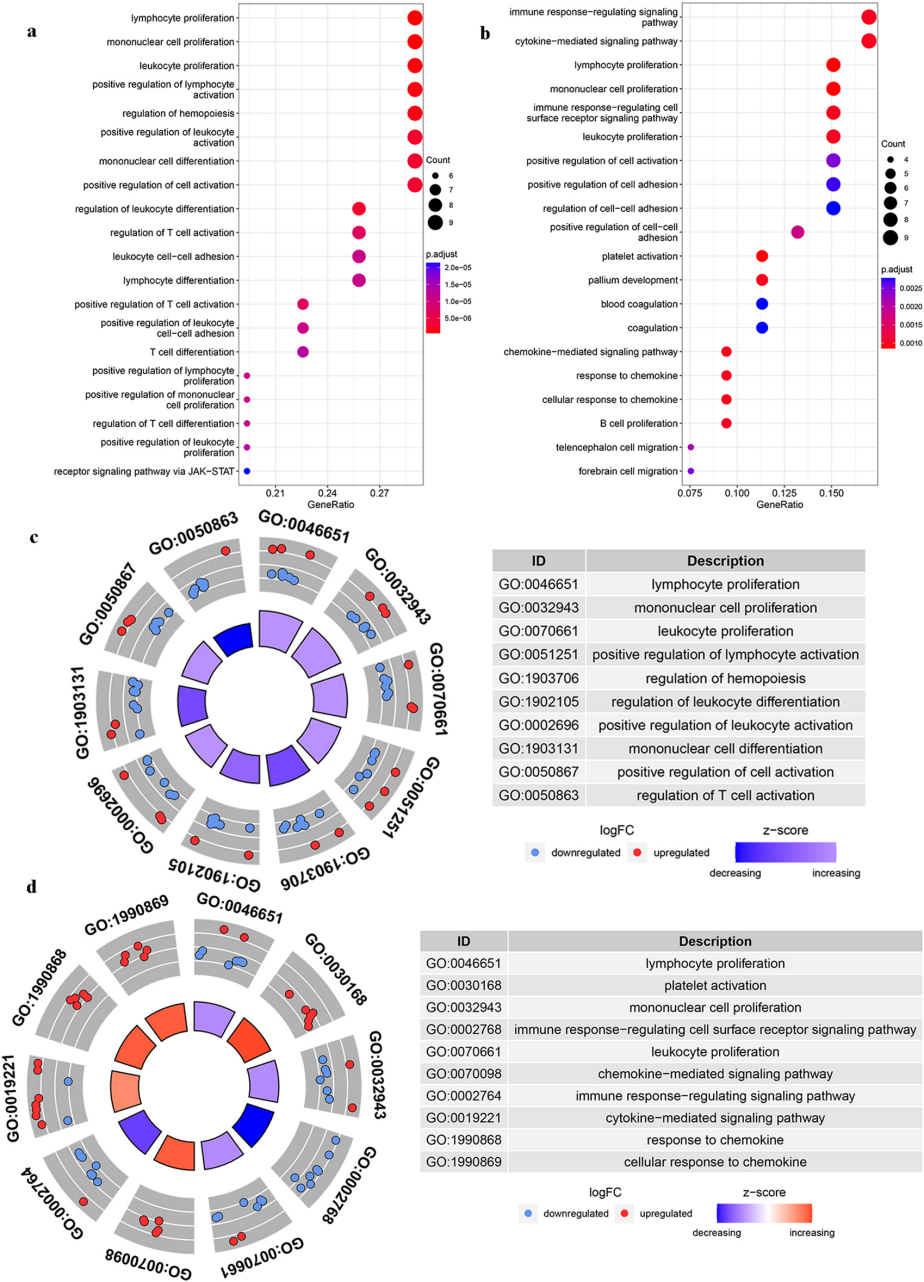
**a** The GO analysis on DEGs induced by the third dose of BBIBP-CorV in elderly groups and the top 20 enriched terms in the biological process were shown. **b** The GO analysis on DEGs induced by the third dose of ZF2001 in elderly groups and the top 20 enriched terms in the biological process were shown. **c** The GO chordal diagram showed the degree of differential expression changes of genes involved in each functional project. Criteria: *p*-value < 0.05. **d** The GO chordal diagram showed the degree of differential expression changes of genes involved in each functional project. Criteria: *p*-value < 0.05

For the purpose of clarifying the functional pathways of the signature DEGs mediated by the third booster vaccine in the elderly group, we conducted GO-term analysis, focusing on the biological process entries of these DEGs. First, we showed the top 20 GO-terms enriched by BBIBP-CorV and ZF2001 in the elderly group in the GO dot-plot (Fig. 5a, b). Coincidentally, DEGs were found to be involved in multiple innate immune pathways, such as lymphocyte proliferation, mononuclear cell proliferation, and leukocyte proliferation. The connection of GO functions with specific DEGs was shown in Figures S1e, f. The chordal diagram was used to further demonstrate the degree of differential expression changes of genes involved in each functional project (Figs. 5c, d). We found that most of the DEGs involved in immune-related functional items were down-regulated, implying that these immune-related pathways were impaired to some extent in the elderly group. This may be a potential reason of early humoral immune deficiency in elderly people.

### The booster vaccine activated PBMC gene expression patterns related to immune responses on day 28

**Fig. 6:**
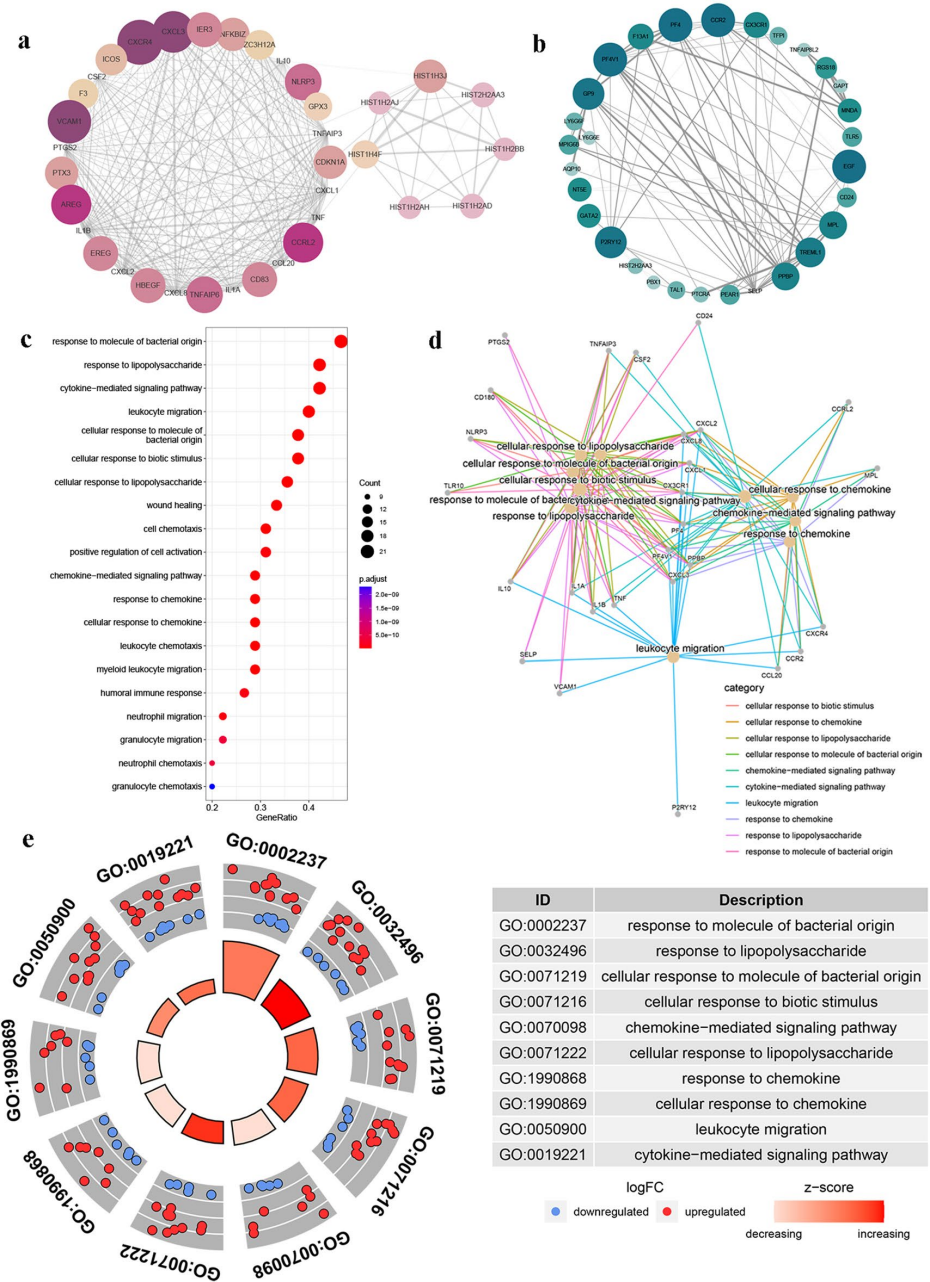
The PPI network extracted from initial PPI networks for the protein products of up and down-regulated DEGs induced by the third dose of BBIBP-CorV in elderly groups after 28 days, consisting of 36 nodes (**a**) and 30 nodes (**b**). **c** The GO analysis on DEGs induced by the third dose of BBIBP-CorV in elderly groups and only the top 20 enriched terms in the biological process were shown. **d** The connection of GO functions with specific DEGs. **e** The GO chordal diagram showed the degree of differential expression changes of genes involved in each functional project. Criteria: *p*-value < 0.05

**Fig. 7:**
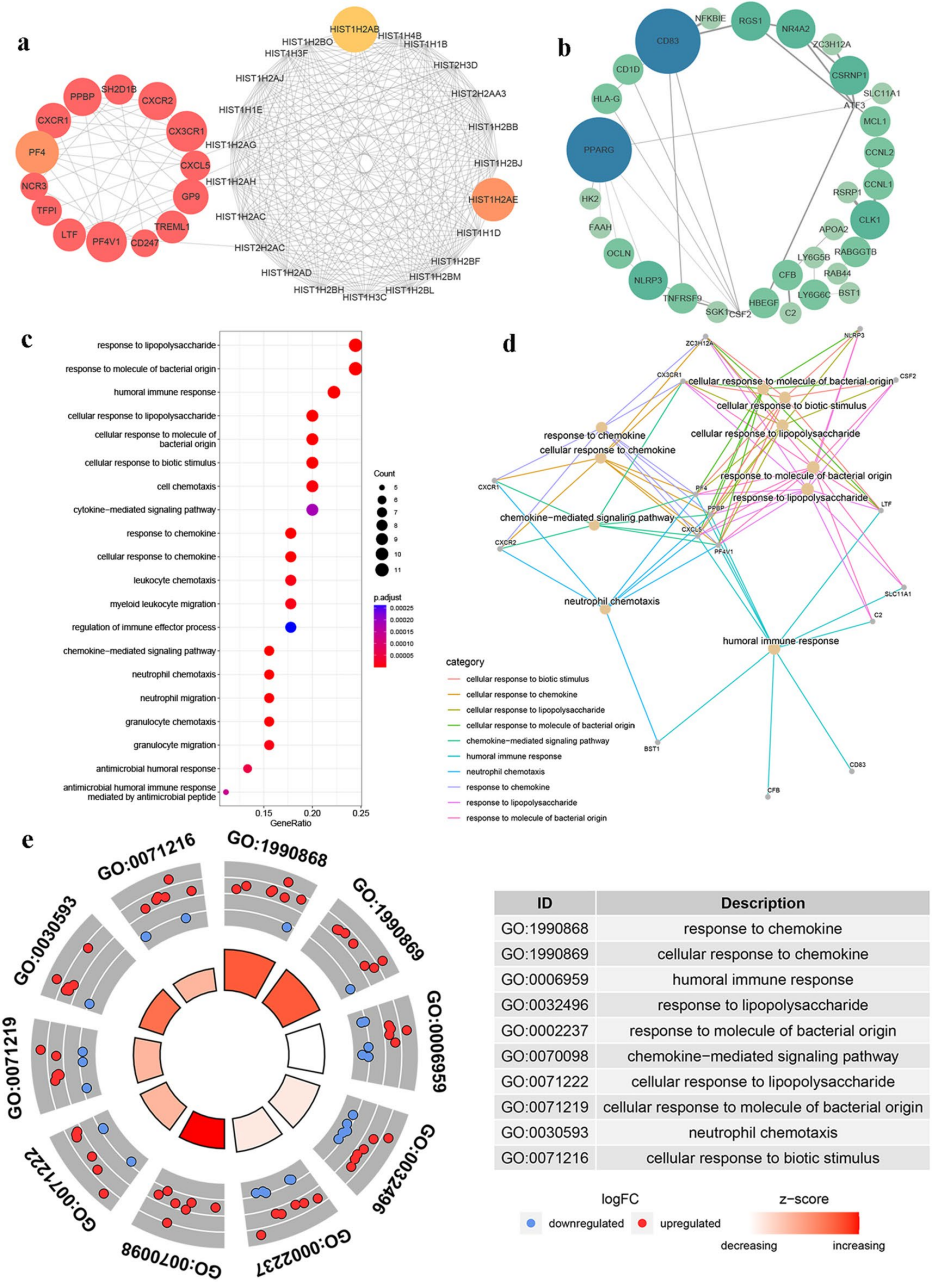
The PPI network extracted from initial PPI networks for the protein products of up and down-regulated DEGs induced by the third dose of ZF2001 in elderly groups after 28 days, consisting of 37 nodes (**a**) and 32 nodes (**b**). **c** The GO analysis on DEGs induced by the third dose of ZF2001 in elderly groups and only the top 20 enriched terms in the biological process were shown. **d** The connection of GO functions with specific DEGs. **e** The GO chordal diagram showed the degree of differential expression changes of genes involved in each functional project. Criteria: *p*-value < 0.05

We similarly used the STRING database and MCODE plug-in of Cytoscape software to prioritize DEGs 28 days after the booster dose. A total of 4 characteristic up-regulated and down-regulated DEG sets were identified induced by the third dose of BBIBP-CorV and ZF2001 in the elderly on Day 28 (Figs. 6a, b and 7a, b).

These characteristic DEGs were also enriched in immune-related biological process GO-terms. Specifically, 28 days after the BBIBP-CorV and ZF2001 booster vaccine, DEGs in the senile group were enriched in several pathways such as the chemokine-mediated signaling pathway and humoral immune response (Figs. 6c and 7c). In the BBIBP-CorV booster vaccine group, these functional entries were mainly executed by CD83, CR2, CXCL1, CXCL2, CXCL3, CXCL8, IL1B, JCHAIN, PF4, PF4V1, PPBP, and TNF, etc. (Fig. 6d). In the ZF2001 booster vaccine group, C2, CXCR1, CXCR2, CX3CR1, CXCL5, CXCR1, CXCR2, LTF, PF4, PF4V1, and PPBP participated in immune regulation (Fig. 7d). Moreover, the chordal diagram further demonstrated that most of the DEGs involved in immune-related functional items were up-regulated, meaning that these immune-related pathways were widely activated 28 days after the booster vaccine in the elderly group (Figs. 6e and 7e).

### The various immune landscapes of age-related gene modules between 7 and 28 days after booster immunization

**Fig. 8:**
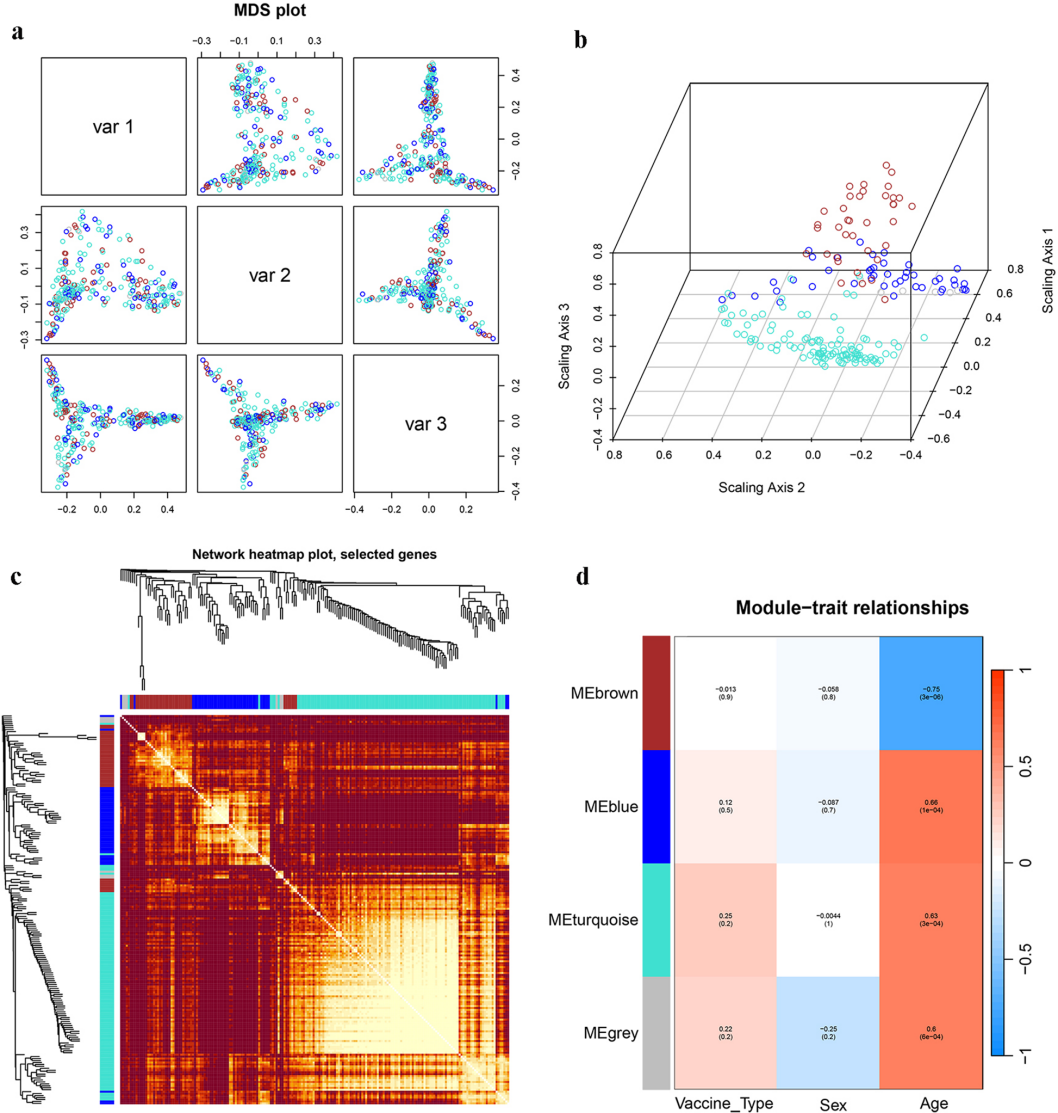
WGCNA of the PBMCs transcriptome after 7 days of the third booster dose. **a**, **b** The 3D cluster map of genes, based on differences in topological overlap matrix (TOM), and assigned module colors. **c** Heatmap depicts the TOM of genes selected for weighted co-expression network analysis. Light color represents lower overlap and red represents higher overlap. **d** Module-trait associations: Each row corresponds to a module eigengene and each column to a trait. Each cell contains the corresponding correlation and p-value

To explore the impact of age characteristics on the transcriptome, we performed an analysis of the transcriptome using weighted gene co-expression network analysis(WGCNA). In order to ensure the construction of a scale-free network, the most suitable β was determined as a soft threshold parameter. A total of four co-expression modules were identified through hierarchical clustering, and a dendrogram of all DEGs was clustered based on a dissimilarity measure (1-TOM) at day 7. These four co expression modules based on differences in topological overlap were displayed in the 3D clustering map and assigned as brown, blue, turquoise, and grey module colors(Fig. 8a, b). Heatmap depicted the Topological Overlap Matrix (TOM) of genes selected for weighted co-expression network analysis(Fig. 8c). For each module, the gene co-expression was summarized by the eigengene and we calculated the correlations of each eigengene with clinical traits, such as vaccine type, gender and age. The correlation between the vaccination signatures and the co-expression module is shown in Fig. 8d, of which the brown module (eigengene value = -0.75, *p* = 0.03 × 10^–6^) was significantly negatively correlated with the age of the participants, but not significantly correlated with vaccine type and gender. The brown module includes 56 genes involved in many immune function-related genes, such as AK5, BTLA, CCR5, CD24C, CR2, DPP4, GCSAM, IL7R, IL23R, LEF1, LTB, RORC, and STAP1, etc. The GO enrichment analysis showed that these genes were mainly involved in "lymphocyte costimulation", "T cell differentiation involved in immune response", "CD4-positive, alpha–beta T cell differentiation involved in immune response", and "T-helper cell differentiation" (Fig. 10a). The above co-expression modules analysis, which is significantly negatively correlated with age, once again indicates that the immune response of the elderly to booster vaccines at 7 days was impaired and delayed.

**Fig. 9:**
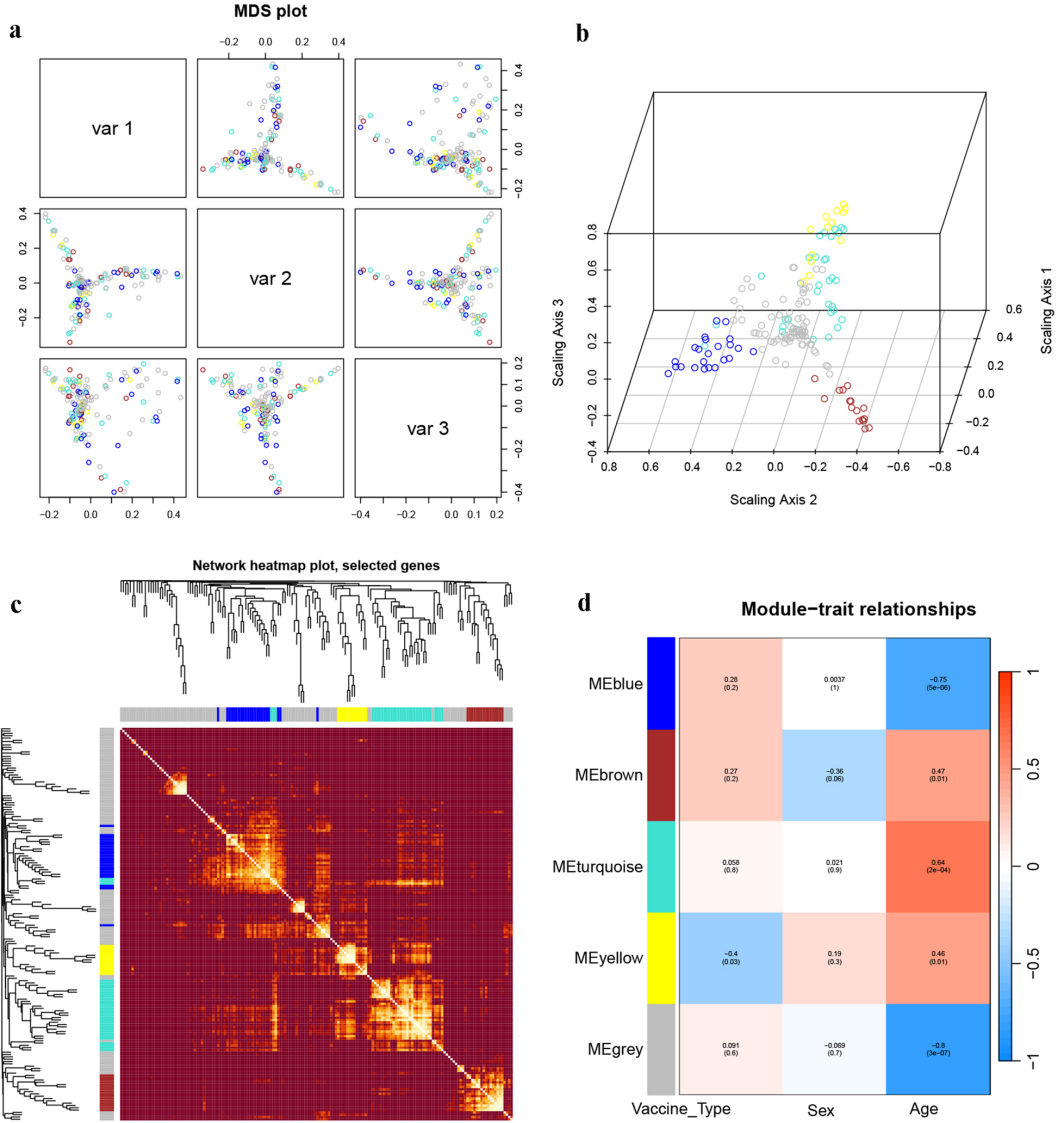
WGCNA of the transcriptome after 28 days of the third booster dose. **a**, **b** The 3D cluster map of genes, based on differences in topological overlap matrix (TOM), and assigned module colors. **c** Heatmap depicts the TOM of genes selected for weighted co-expression network analysis. Light color represents lower overlap and red represents higher overlap. **d** Module-trait associations: Each row corresponds to a module eigengene and each column to a trait. Each cell contains the corresponding correlation and p-value

**Fig. 10:**
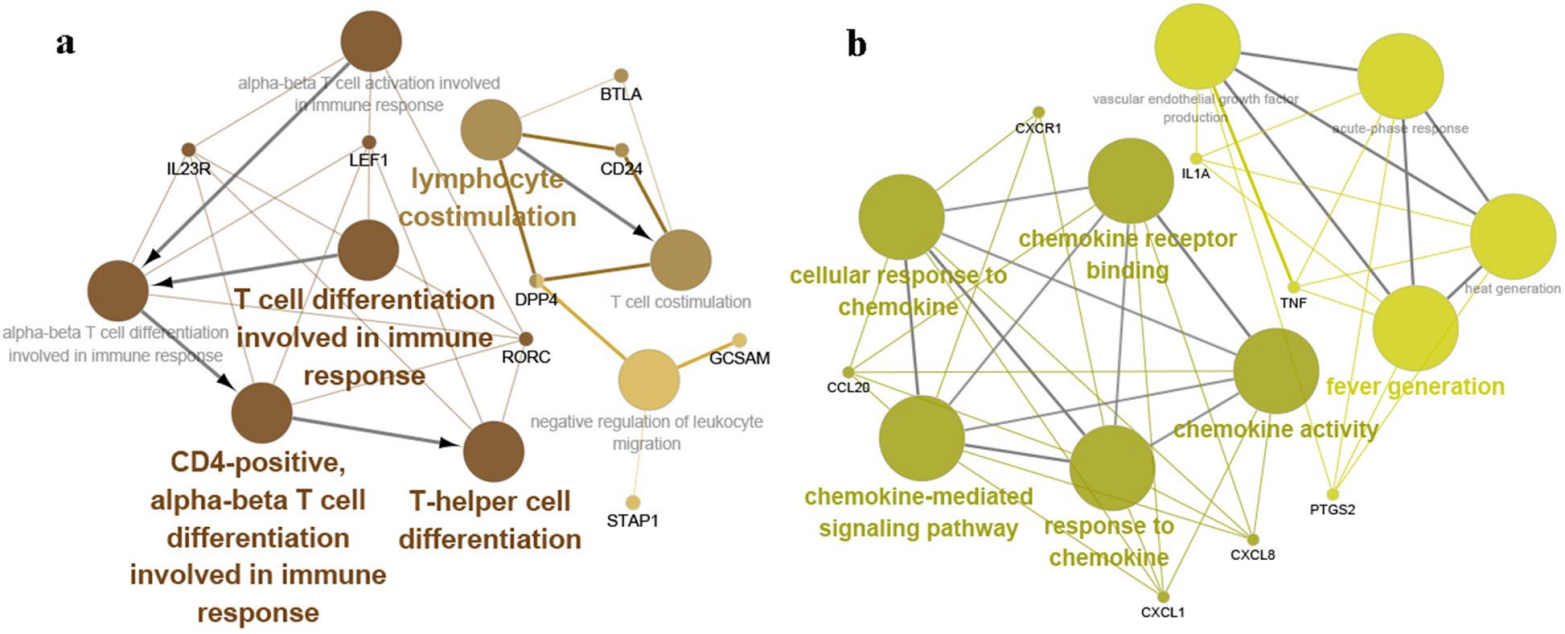
**a** The GO functional enrichment analysis of brown gene module after 7 days of the third booster dose. **b** The GO functional enrichment analysis of brown and yellow gene module after 28 days of the third booster dose

A similar WGCNA was performed on transcriptome data at 28 days. Five co-expression modules were identified on day 28, of which the brown module (eigengene value = 0.47, *p* = 0.01) and yellow module (eigengene value = 0.46, *p* = 0.01) were significantly positively correlated with the age of the participants (Fig. 9a-d). The co-expressed genes of these two modules are mainly involved in "fever generation", "chemokine activity", "chemokine receptor binding", "response to chemokine", and "chemokine-mediated signaling pathway" (Fig. 10b). This means that after 28 days of the booster vaccine, the immune transcriptional landscape associated with the elderly significantly improved.

## Discussion

As the COVID-19 pandemic has refocused attention on the vulnerability of elderly adults to emerging infectious diseases, it is critical to examine the response of elderly adults to the third booster dose and to discuss the impact of aging on immunity and vaccination. Systematic biology studies, which combine conventional immunological approaches and transcriptional landscapes, have become a key approach for a comprehensive understanding of the immune response to vaccination at the molecular level [16, 17]. This study used a systematic biology approach to assess specific antibody levels and transcriptional response profiles to the third booster dose in elderly participants over 70 years of age. Overall, our data showed a tardive but effective antibody response, and variable immune-related transcriptional footprints in the elder adults compared to younger participants.

Both in the clinical setting and in research, the response to vaccination is most commonly assessed by the levels of antibodies within circulation [18]. Our study found that IgG antibody responses in elder adults were lower after 7 days of vaccination, but after 28 days of vaccination, they could achieve similar levels of characteristics to those in younger adults. Similar delays in the antibody response have been observed in previous surveillance of other vaccines, including attenuated yellow fever vaccines [19] and primary inactivated hepatitis B vaccine [20]. This delayed antibody response may be caused by age-related declines in innate and adaptive immune responses. Interestingly, several recent studies of the COVID-19 vaccine are consistent with antibody results that we monitored after 28 days of vaccination, with no striking difference in age-related antibody responses [21, 22]. In a one-center study involving 4970 volunteers, it was found that IgG levels were multiple highs in older individuals evacuated with mRNA-1273 than BNT162b2 [23]. And elderly adults could still develop a robust antibody response to the third dose [24, 25].

The development of high-throughput technologies has allowed vaccinologists to study vaccine-induced immune responses in greater depth than ever before [16]. The down-regulated characteristics DEGs of BBIBP-CorV homologous boost and ZF2001 heterologous boost in the elder group on day 7 were enriched in many innate immune pathways. The impairment of immune-related transcriptional responses has also been observed in the elderly after vaccination with other vaccines. For example, the response characteristics of the elderly are dysregulated after the influenza vaccine [26–28]. The immune response to the hepatitis B vaccine was also impaired in the elderly [20, 29]. However, 28 days after the booster vaccine, "response to chemokine", "chemokine-mediated signaling pathway", "leukocyte migration", and "humoral immune response" were widely activated in the elderly group. Given the critical role of the innate immune response in regulating the strength, quality, and duration of the later adaptive immune response [30], the characterization of innate immune activation can, in part, predict whether a vaccine will induce appropriate protection [31]. The transcriptional impairment and activation of immune responses 7 and 28 days after the booster vaccination may be the underlying mechanism leading to significant changes in the antibody response in the elderly group.

The WGCNA helped to identify key molecular and cellular features of protective immunity that were significantly associated with sample characteristics. Our results show that 7 days after the COVID-19 booster vaccination the age of participants was significantly negatively correlated with the modules involved in functional pathways related to T helper cell stimulation, activation, and differentiation. T helper cells (CD4 T cells) are one of the important subsets of T cells [32], and play a central role in the induction and regulation of adaptive immunity [33]. The impaired CD4 T cell population dynamics may also be the reason for the hyporesponsiveness in elderly on day 7. However, after 28 days of vaccination, chemokine-related gene co-expression modules were significantly positively correlated with age. These functions are mainly performed by some immune-related up-regulated hub genes, including CCL2, CXCL1, CXCL8, CXCR1, IL1A, TNF, and PTGS2. Chemokines play an important role in the inflammatory response by attracting leukocytes to the site of infection through their strong chemotactic ability and are crucially involved in the regulation and maintenance of immune responses [34]. The role of chemokine/chemokine receptor systems in vaccines needs further investigation.

The limitations of the study are that it did not directly explore the subpopulation frequencies of PBMCs, nor did not delve into the mechanisms of cellular immunity, which also play an important role in evaluating the efficacy of vaccines. In addition, due to the short follow-up time, the durability of the third dose of the COVID-19 vaccine needs further study.

## Conclusion

In conclusion, we used a systematic biology approach to comprehensively characterize the molecular network of vaccine-driven early innate and adaptive immunity to prospectively predict the immune response to a third booster dose in elderly adults over 70 years of age. These results reveal the potential adverse effects of immunosenescence on the effectiveness of vaccination at 7 days, observed encouraging antibody and transcriptional responses at 28 days, and clearly underscore the benefits of a third dose.

## Materials and methods

### Study population and sample isolation

In November 2021, we recruited healthy SARS-CoV-2-naïve individuals who had completed two doses of inactivated vaccine for more than 6 months to participate in this study in Liaocheng City, Shandong Province. All participants were assigned according to age characteristics to a younger group (age < 70) and an elder group (age ≥ 70) for a third homologous boost with BBIBP-CorV or heterologous boost with ZF2001. The anticoagulant and procoagulant venous blood samples were collected 7 and 28 days after booster vaccination, and serum and peripheral blood mononuclear cell (PBMC) samples were isolated for further study.

### Quantitative SARS-CoV-2 IgG detection

The IgG antibodies were detected using an indirect ELISA kit (Vazyme, China) based on the spike protein of SARS-CoV-2. All serum samples were diluted to a concentration gradient of 1:200, and optical density (OD) was read at 450 nm and 630 nm after incubation, enzyme labeling, chromogenic reaction, and termination steps. The specific operation was carried out in strict accordance with the instructions. The standard curve was prepared with 6 standard substances with known antibody concentrations provided in the kit, and the OD value of the sample to be tested was converted to the antibody concentration. The antibody concentration of the IgG antibody was calculated after three repetitions. The mean with SD was used to describe antibody titers and statistical significance was analyzed by unpaired t-tests with log-transformation using GraphPad Prism 8.0.

### Transcriptome sequencing

The transcriptome high-throughput sequencing of PBMC samples from participants was performed according to our previous research methods [35]. Total RNA was extracted from PBMCs by using the RNeasy Mini Kit (Qiagen, Germany) according to the manufacturer’s instructions. The concentration and integrity of total RNA were checked using the Qubit RNA Assay Kit in a Qubit 4.0 Fluorometer (Life Technologies, USA) and the RNA Nano 6000 Assay Kit of the Bioanalyzer 2100 System (Agilent Technologies, USA) respectively. A total amount of 100 ng total RNA per sample was used to prepare the rRNA-depleted cDNA library by Stranded Total RNA Prep Ligation with Ribo-Zero Plus Kit (Illumina, USA). The final library size and quality were evaluated using an Agilent DNA 1000 Kit (Agilent Technologies, USA), and the fragments were found to be between 250 and 350 bp in size. The library was sequenced using an Illumina NextSeq 2000 platform to generate 100 bp paired-end reads.

### Differentially expressed gene (DEG) identification

The quality control(QC), trimming, and mapping of the RNA-seq raw fasta data to the human reference genome hg38 were performed in the CLC Genomics Workbench. The gene expression level was measured based on the transcripts per million (TPM). We calculated normalization factors using iterative edgeR [36] and limma [37] packages, and the standardization and filtering of gene expression were accomplished by the voom, lmFit, and eBayes functions. DEGs were filtered out according to *p*-value < 0.05 and 2^logFC_cutoff criteria, and visualized as volcanoes and heatmaps by the pheatmap package in R (version 4.1.0).

### Protein protein interaction (PPI) network construction

In order to explore the relationship between proteins encoded by identified up and down-regulated DEGs, the initial PPI networks for the protein products of DEGs were constructed using the STRING Database (version 11.5) [38], and then the network was visualized and analyzed with Cytoscape software (version 3.8.28) [39]. The molecular complex detection (MCODE) plugin in Cytoscape software was used to screen the characteristic DEGs from the initial PPI networks.

### Pathway enrichment analysis

To be aware of the prospective functions of characteristic DEGs identified by the PPI network analysis, Gene Ontology (GO) terms were identified using the clusterProfiler 4.0 package [40]. *P* < 0.05, subjected to Bonferroni adjustment, was defined as the cut‑off criterion. Data visualization was performed using the ggplot2, enrichplot, GOplot, topGO, circlize, and ComplexHeatmap packages in R (version 4.1.0).

### Identification of age-related modules in adults with the third dose

To identify gene modules related to the age characteristics in the COVID-19 booster vaccine, we performed weighted gene co-expression network analysis based on transcriptional profiles and sample characteristics through the WGCNA package [41] in R software. First, the best β value was confirmed with a scale-free fit index larger than 0.85 as well as the highest mean connectivity by performing a gradient test from 1 to 30. Subsequently, the topological overlap matrix (TOM) transformed by the adjacency matrix was then clustered by dissimilarity between genes, and we performed hierarchical clustering to identify modules. Finally, the co-expressed genes were determined by calculating the module membership (MM) and gene significance (GS) of the genes in the target modules. The immune landscape of important modules was analyzed using the ClueGO plugin [42] of Cytoscape software (version 3.8.2). *P* < 0.05, subjected to Bonferroni adjustment, was defined as the cut − off criterion.

### Supplementary Information

**Additional file 1: Figure S1. **The PPI network extracted from initial PPI networks for the protein products of up and down-regulated DEGs induced by the third dose of BBIBP-CorV in elderly groups after 7 days, consisting of 21 nodes(a) and 25 nodes(b). The PPI network extracted from initial PPI networks for the protein products of up and down-regulated DEGs induced by the third dose of ZF2001 in elderly groups after 7 days, consisting of 34 nodes(c) and 42 nodes(d). (e)The connection of GO functions with specific DEGs induced by the third dose of BBIBP-CorV in elderly groups after 7 days. (f)The connection of GO functions with specific DEGs induced by the third dose of ZF2001 in elderly groups after 7 days.

**Additional file 2: Table S1. **The characteristics of 36 participants boosted by BBIBP-CorV.

**Additional file 3: Table S2. **The characteristics of 35 participants boosted by ZF2001.

## Data Availability

The RNA-seq data of this study has been uploaded to the GEO database (NCBI) under accession numbers GSE206023 and GSE234008, and will be made available to others with publication.
