# Supplementary figures and images for: Antibody and transcription landscape in peripheral blood mononuclear cells of elderly adults over 70 years of age with third dose of COVID-19 BBIBP-CorV and ZF2001 booster vaccine
Source: Immun Ageing. 2024 Jan 27;21:11. doi: 10.1186/s12979-023-00408-x (PMC10821575; doi:10.1186/s12979-023-00408-x)

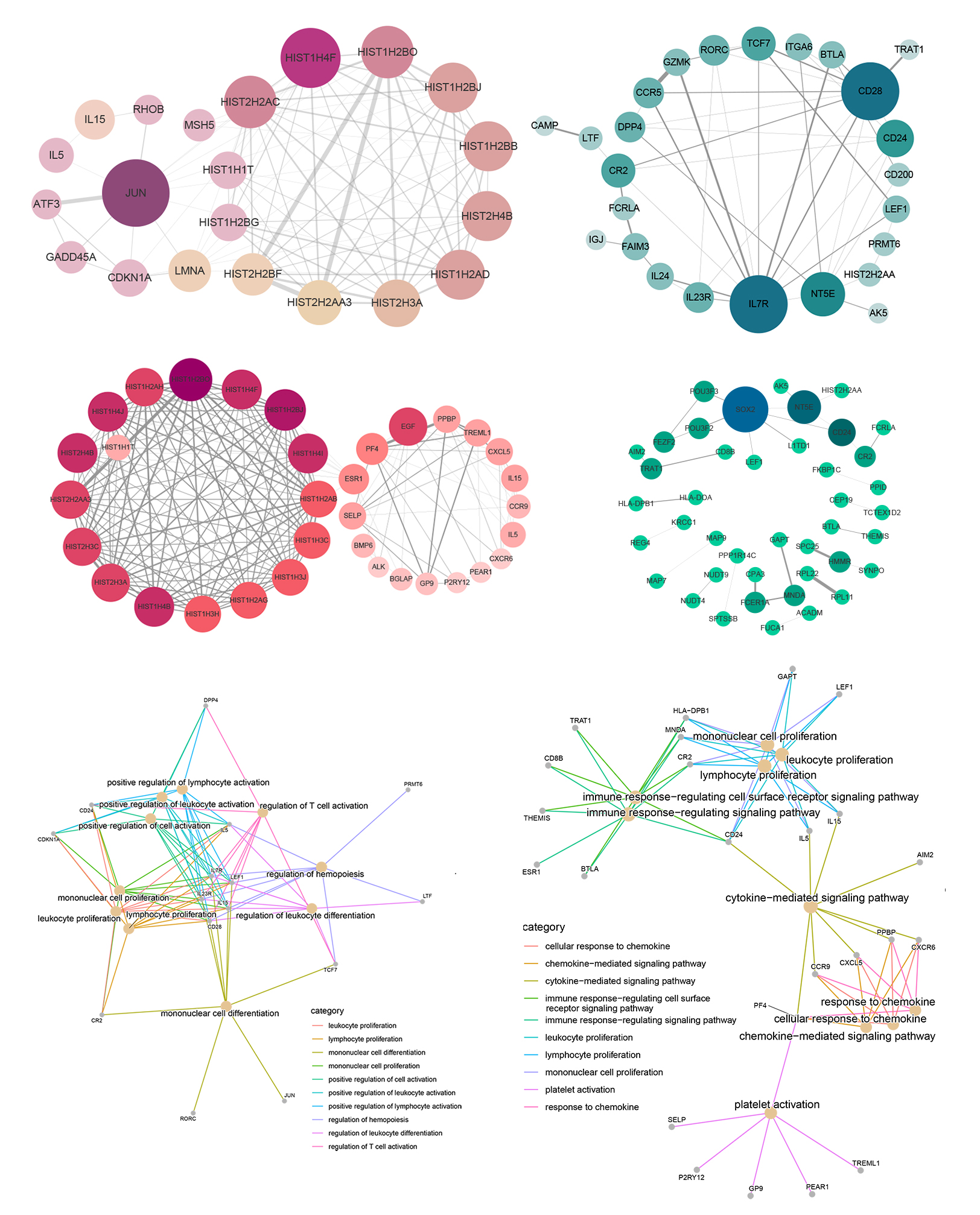

Supplement: Supplementary file 1 — Additional file 1: Figure S1. The PPI network extracted from initial PPI networks for the protein products of up and down-regulated DEGs induced by the third dose of BBIBP-CorV in elderly groups after 7 days, consisting of 21 nodes(a) and 25 nodes(b). The PPI network extracted from initial PPI networks for the protein products of up and down-regulated DEGs induced by the third dose of ZF2001 in elderly groups after 7 days, consisting of 34 nodes(c) and 42 nodes(d). (e)The connection of GO functions with specific DEGs induced by the third dose of BBIBP-CorV in elderly groups after 7 days. (f)The connection of GO functions with specific DEGs induced by the third dose of ZF2001 in elderly groups after 7 days. [file 12979_2023_408_MOESM1_ESM.jpg]
